# Modern Diets and the Health of Our Planet: An Investigation into the Environmental Impacts of Food Choices

**DOI:** 10.3390/nu15030692

**Published:** 2023-01-30

**Authors:** Kiera A. Dixon, Malia K. Michelsen, Catherine L. Carpenter

**Affiliations:** 1 Department of Environmental Health Sciences, Fielding School of Public Health, University of California at Los Angeles, Los Angeles, CA 90024, USA; 2 Institute of the Environment and Sustainability, University of California at Los Angeles, Los Angeles, CA 90024, USA; 3 UCLA Center for Human Nutrition, David Geffen School of Medicine, University of California at Los Angeles, Los Angeles, CA 90095, USA

**Keywords:** modern diets, health, nutrition, carbon footprint, climate change, environmental concerns, sustainability, sustainable diet, Mediterranean, keto, paleo, vegan, climatarian

## Abstract

Popular modern diets are often seen as a panacea for improving health and promoting weight reduction. While there is a large body of literature reporting the health benefits of popular diets, few studies have described their planetary benefits. Our investigation aims to evaluate the simultaneous impacts of six popular diets within the United States on both human and planetary health. Using carbon footprint databases and representative meal plans, the environmental and health-related impacts of the Standard American, Mediterranean, vegan, paleo, keto, and climatarian diets are compared using the currently available literature. Results indicate that diets that exhibit lower carbon footprints also have positive effects on human health. The diets found to have the lowest environmental impacts were the vegan, climatarian, and Mediterranean diets. These low-carbon-footprint diets can likely be attributed to a reduced reliance on ruminant meat (cattle and sheep) and processed food consumption, while diets with high carbon footprints are more dependent on ruminant meat and saturated fat. Moderate consumption of meats such as chicken, pork, and fish in conjunction with an emphasis on locally grown fruits and vegetables can be maintained without adversely affecting the planetary carbon footprint and with the added benefit of promoting good health. Thus, making simple substitutions within each individual’s diet can be advertised as an effective approach to collectively lower the environmental impact in tandem with improving health and longevity.

## 1. Introduction

People within the United States (U.S.) have been making dietary choices based on health claims and ethical considerations for generations. It is becoming increasingly apparent that the impact of one’s diet does not simply end at their health, as there are broader, planetary impacts of food choices. One of these planetary impacts is measured by carbon footprints. The carbon footprint of an item is generally defined as “the amount of greenhouse gas and, specifically, carbon dioxide emitted by something (such as a person’s activities or a product’s manufacture and transport) during a given period” [1]. However, a generally acceptable and consistent academic definition has yet to be agreed upon [2]. In terms of food, carbon footprints depend on the source and composition of each food item. Each stage in the supply chain contributes to differential emissions, ranging from land-use changes, farms, and animal feed, to transportation, retail, and packaging. These emissions add up quickly; in the U.S., food is responsible for 26% of total greenhouse gas emissions [3]. This statistic is not widely known by Americans; but, if it were, it may affect their decision-making on what they consume [4]. The EAT-Lancet Commission produced guidelines for following a flexitarian diet that benefits human health and the environment. Healthy diets were described to have “an appropriate caloric intake, a diversity of plant foods, low amounts of animal source foods, contain unsaturated rather than saturated fats, and limited amounts of refined grains, highly processed foods and added sugars.” With this dietary framework, food systems can potentially provide this flexitarian diet “for an anticipated world population of nearly 10 billion people by 2050 and still stay within a safe operating space on Earth. However, even small increases in red meat or dairy foods would make this difficult or impossible” [5].

The purpose of our study is to bring attention to the inextricable link between human health and the environment by contrasting carbon-footprint values with the known health benefits of popular diets. Five common diets were selected for comparison: Standard American, Mediterranean, vegan, paleo, and keto. Also included was the climatarian diet—one whose primary goal is to reduce the environmental impact of one’s dietary choices [6]—for a total of six diets that we compared. This study investigates differences in both the carbon-footprint effects and the health effects associated with each. 

Carbon-footprint information can not only assist consumers but also food brands that already have climate in mind during production. Although processed foods often have higher footprints than their whole-food counterparts, they do not always have to. Some food producers are adjusting their farming and production processes with climate in mind. Several food producers have focused their production on foods that reduce their carbon footprints, including Airly Foods [7], Moonshot Snacks [8], Quinn Snacks [9], Alter Eco [10], and Patagonia Provisions [11]. For example, a box of Airly’s Oat Clouds cheddar crackers removes 21 g of CO_2_ from the air [7]. Simple food choices to replace traditional crackers with climate-friendly snacks, like this, can help reduce the carbon footprint. These foods that are developed with climate change in mind illustrate a growing trend in the market appeal of sustainably produced food and food commodities.

The purpose of our study was to evaluate food choices that represent consumption patterns found in popular diets and their potential impacts on climate change. We also evaluated the impacts of these diets on human health to provide an understanding about whether diets that are healthy and promote disease prevention also contribute toward a reduction of the environmental burden required for food production. While all diets require resources from the environment for food production and transportation, some may be more impactful than others.

## 2. Methods

The goal of our study was to calculate the environmental impacts of six diets (Standard American, Mediterranean, vegan, paleo, keto, and climatarian) by comparing the carbon emissions associated with the ‘typical day’ of dining that characterizes each of the six diets. The study leverages existing information as opposed to calculating the emissions for each food item directly. There are several databases available in peer-reviewed scientific articles that collate carbon emissions from food production. This study relies on two of them. First is the SU-EATABLE LIFE (SEL) database [12,13], a comprehensive carbon and water index that consolidates both a large number of recently published and peer-reviewed articles derived from the literature as well as previous work compiled via Clune, et al. [12]. SEL was chosen as a reliable dataset as a result of to its extensive breadth, low cost, accessibility, and recent publication. The value of each food item was additionally provided in the desired functional unit (kg CO_2_-eq), and “the system boundary considered in the SEL database is the distribution center to consumers located in the country of origin [and] hence excludes post-market phase like for example cooking” [13]. This was important for the purposes of this research paper, because the goal was to analyze the individual carbon footprints of the specific food items in each diet from their initial cultivation or production processes to their market arrivals. Thus, the inclusion of transportation was not essential in the decision to use SEL in our research process.

Of the 3349 food commodities available for use in the SEL database, we narrowed our range to those from the region of North America (including the U.S. and Canada only). This resulted in a much smaller database composed of 299 different food items, 212 of which were from the U.S. The value of limiting our scope to North America instead of just the U.S. lay in the 87 additional food items gained from the inclusion of Canada, allowing for an increase in the diversity of food items available and decreasing the need for a secondary database for the sake of consistency within system boundaries.

Some food items included in the meal-plan scenarios were not available via SEL. Thus, a secondary database was sourced to fill in the gaps [14]. The work of Song et al., “Large-Scale Microanalysis of U.S. Household Food Carbon Footprints and Reduction Potentials,” provides an additional 83 food commodities. While the SEL dataset is sourced from the larger and accredited EcoInvent database, our geographical limitations warranted the supplementation of a second data source [14]. Apart from their focus on the U.S., this secondary database shares similarities with SEL in that “post-farm-gate activities such as packaging and transportation are not included due to lack of data,” meaning that there were no overestimations in the carbon footprint caused by using two separate databases in which one included transportation and the other did not, and no additional conversions were required.

There were two exceptions to this approach. First, no U.S. or Canadian values for avocado (a staple food in both the keto and vegan diets) existed in the database, so avocado of Mexican origin was used from the SEL. Second, on occasion, there were opportunities to utilize certain brands of foods that provide their public footprint calculations directly, such as Airly Cheddar Oat Cloud Snack Crackers as a snack item in the climatarian meal scenario.

The databases were used to quantify the carbon emissions of representative, single-day meal plans. The exceptions to this were the Airly food items, which are found as snacks in the climatarian diet. Airly performed its own life-cycle assessments of their food items, which included a net 21 g CO_2_-eq removed from the atmosphere per box of Cheddar crackers [7]. Representative meals and their respective nutrient and carbon-footprint values are found in the Supplemental Material and are summarized in Table S2.

Nutritional data regarding the kilocalorie, carbohydrate, fat, protein, and fiber content and food weight of each meal were retrieved from the U.S. Food and Drug Administration (USDA) FoodData Central database [15]. Each of the six meal plans was standardized to 2000 calories (±200 kcal) for alignment with the daily average caloric recommendations of the USDA, which is used on most nutrition labels [15]. However, this 2000-calories-per-day recommendation is not meant to apply to everyone, and it is acknowledged that the total number of calories a person needs each day varies depending on a number of factors, namely the person’s age, sex, height, weight, level of physical activity, and pregnancy or lactation status [16]. 

In many countries, average calorie consumption exceeds the 2000-calorie recommendation. According to the United Nations Food and Agriculture Organization (FAO), the average American consumed around 3782 calories per day in 2018—almost double the amount that is being examined for this study [17]. While many Americans do not appear to be concerned about their high caloric intake, the standard meal plans were constructed to optimize health rather than for satiety or pleasure. In addition, each diet relied upon an associated organization, institution, or author to provide an ideal meal plan for inspiration: the Standard American diet was adopted from MyPlate.gov [18], Mediterranean from the PREDIMED protocol that was modified for the Southern Californian population [19], keto from Neudorf et al. [20], paleo from both The Nutrition Source [21] and Jönsson et al. [22], climatarian from climatarian.com [6], and vegan from Hever [23].

## 3. Results

### 3.1. Diet Backgrounds

Six common diets within the U.S. were selected for comparison: the Standard American, Mediterranean, vegan, paleo, keto, and climatarian diets. These diets differ from one another on a number of factors. Each diet’s following within the U.S. ranges from 3 million to 260 million [24] followers out of a total estimated 332 million people as of 2021 [25] and is subject to fluctuation. Some diets have flexibility in ‘allowable’ foods, while others have more rigid rules to follow in order to maintain the diet. Health impacts range across diets and their respective nutrient compositions from beneficial to controversial, with some even considered harmful. Their environmental impacts can be organized into two categories: high impact and low impact, with respect to their carbon emissions. The following are descriptions of each diet in relation to their general criteria, health benefits, and environmental considerations (Table 1).

#### 3.1.1. Standard American

The Standard American diet, more technically described as the “Western Diet” in academic and scientific literature, is less of an intentional meal plan and is commonly focused on food for sustenance instead of the prioritization of health [26]. The Standard American diet is included in this paper as a control or basis from which to compare the other diets. The Western Diet rose to prominence as a meal plan (and has been continually expanding in reach) beginning in the 1990s. Exponential economic growth and the globalization of western food supply chains had led to the expansion of modern industrial manufacturing in the food industry, giving way to a new standard for access and distribution [27]. Many of these foods included synthetic sweeteners, engineered oils, and syrups that would come to dominate the newest foods in the market, becoming staples in what is now coined as the diet of the Standard American. Few would state that they adhere to the “Standard American diet,” so one must approximate the number of followers. The Center for Disease Control and Prevention (CDC) reported that “in 2015–2018, 17.1% of U.S. adults aged 20 and over were on a special diet on a given day” [28]. With this fact in mind, and for the purposes of this paper, we assume that the vast majority of the U.S. population (over 80%) are not practicing special diets and follow what could be characterized as the Standard American diet. 

**Table 1:** Summary of Diet Backgrounds.

Diet	Present Following in U.S. (Estimated)	General Criteria	Health Benefits	Environmental Considerations
Standard American	80% (260 M) [29]	Meals with five food categories (grains, protein, dairy, fruits, vegetables, and oils on the side). No restriction of salt, sugar, saturated fats, or processed foods.	Variable. Balanced diets that focus on whole foods show a reduced risk of disease, while imbalanced and heavily processed diets show the opposite effect.	Higher impact
Mediterranean	5% (16 M) [24]	Emphasizes whole grains, fruits and vegetables, nuts, seeds, legumes, fish, and olive oil, as well as meat, dairy, egg products, and red wine in moderation. Excludes processed foods and “bad” fats.	Reduced risk of cardiovascular disease, stroke, diabetes, and cancer.	Lower impact
Vegan	5% (16 M) [24]	Excludes animal-based products.	Variable. Balanced diets that focus on whole foods show a reduced risk of disease, while imbalanced and heavily processed diets show the opposite effect.	Lower impact
Paleo	3% (10 M) [24]	Excludes processed foods, refined/artificial sugars, salt, grains, legumes, and most dairy products.	Generally positive impact on health and reduced disease risk.	Higher impact
Keto	5% (16 M) [24]	Emphasizes foods rich in fat and protein. Significantly limits carbohydrates such as sugars and grains.	Individual variation, although general success in short-term weight loss.	Higher impact
Climatarian	Unknown	Emphasizes local, seasonal, and fresh food that requires minimal transportation, refrigeration, and processing. Limits pork, poultry, and sustainable fish consumption. Excludes ruminant meats (lamb, goat, beef).	Unknown, although similarities in composition to the Mediterranean diet.	Lower impact

The eating habits of many Americans are often described as lacking in adequate and substantial portions of fruits, vegetables, and whole grains coupled with an excess of processed foods and a heavy emphasis on salt, sugar, and saturated fats [28]. Thus, the diet is inherently deficient in many of the valuable micronutrients, macronutrients, vitamins, and minerals necessary for optimal health. Furthermore, western food practices often seek “highly palatable foods that can trigger eating addictive-like behaviors” (which are often satisfied by highly processed or pre-packaged food items) and result in many preventable diseases such as “atherosclerosis, type II diabetes, hypertension, and, [most commonly], obesity” [30,31]. The well-established and increasingly prominent obesity epidemic within the U.S.—in 2018 the obesity prevalence was found to be 41.9% in the U.S. population of adults—is proof enough of the damage that can be caused by a calorically rich yet nutrient-poor dietary regimen [32]. The Western Diet is not typical in its intention as a deliberate and actual diet in the traditional meaning of the word. Thus, it is not surprising that there are no apparent health benefits for those that subscribe to this particular style of eating. In fact, the elevated intake of highly processed food items combined with low quantities of whole foods only seems to coincide with or result in a multitude of adverse health effects such as heart disease, metabolic and gastrointestinal disturbances, neurodegenerative diseases, diabetes, and infertility, to name but a few [33]. Furthermore, because the Western Diet is widely considered to be notoriously unhealthy, those that follow the American regimen are less likely to engage in daily physical activity and are more likely to engage in the consumption or recreational use of alcohol and drugs, which lead to diseases such as coronary heart disease, diabetes, obesity, and depression [34,35]. 

The meal-plan scenarios outlined for the purpose of this research paper were chosen to optimize health, and thus will not include as many processed foods as may be considered typical for the average American. In order to combat the epidemic of diet-induced illness, the U.S. Department of Agriculture developed MyPlate^®^ to educate young children and adults on the five food groups that make up the ideal food pyramid [18]. Government-sponsored programs such as those that aim to demonstrate the importance of building balanced proportioned meals are filled with each of the five food categories (fruits, vegetables, grains, protein, and dairy) and oils that are included on a plate [36].

In regards to the environmental footprint of the Standard American diet, it is considered to be quite high as a result of its emphasis on the daily consumption of meat and dairy, two food items that have the highest greenhouse gas emissions; its land-use activity; and its acknowledged environmental impact(s) in each of the industries’ respective production processes [37]. In addition, the demand for highly processed foods is itself an energy-intensive manufacturing process, requiring an increased energy input (in the form of fossil fuels) to meet said demand. The intake of pre-packaged and processed foods also produces excessive plastic pollution. To serve as a comparison for the other considered diets, nutrient requirements for typical meals following MyPlate were used to represent the Standard American diet with an understanding that the lack of processed foods may represent an underestimation of the environmental impact from an ‘average’ American diet.

#### 3.1.2. Mediterranean

The Mediterranean diet first gained international popularity in the 1980s as the medical community began to note the long life expectancies and low risks of cardiovascular disease in people who resided in Mediterranean Basin regions, such as Crete and Southern Italy [38]. In the U.S., an estimated 5% of the population currently follows the Mediterranean diet [24].

Less of a diet and more of a lifestyle, the Mediterranean diet has no hard and fast rules or restrictions but rather emphasizes a focus on whole grains, fruits and vegetables, nuts, seeds, legumes, fish, and olive oil. It does include meat, dairy, egg products, and even red wine in moderation; however, consumption of these foods tends to be lower than in the Standard American diet [39,40]. The traditional Mediterranean diet tends to avoid heavily processed foods as well as desserts. 

The main difference that sets the Mediterranean diet apart from others is its focus on monounsaturated “good fats,” namely nuts and olive oil, that lower low-density lipoprotein cholesterol—the “bad” cholesterol [41]. Studies have shown that consumption of monounsaturated fatty acids (MUFAs) is associated with many health benefits, including a reduction in the risk of cardiovascular disease, stroke, diabetes, and cancer and an improvement in mental health [19,42]. The results of randomized trials found that those in the Mediterranean diet intervention had lower risks of heart attack, stroke, and death from cardiovascular disease than the control group [37,43]. In addition, the Mediterranean dietary intervention group had a lower overall mortality rate than other comparison groups and a lower overall cancer mortality rate in a recent meta-analysis of the Mediterranean diet and cancer [44]. The lack of evidence for any negative health effects from this diet [45], combined with the extensive research on its health benefits, has promoted the general public to recognize the Mediterranean diet as one of the healthiest diets to follow.

The Mediterranean diet also has a comparatively favorable impact on the environment. The carbon footprint of the standardized Mediterranean diet was lower than that of the Standard American, keto, and paleo diets. These three diets include high intakes of red meat and dairy products known to elicit high greenhouse gas emissions and cause excessive land use by the beef and dairy industries. Low consumption of processed foods in the Mediterranean diet also decreases plastic consumption and emissions associated with the processing, packaging, and transportation of these products [46]. The guidelines of the Mediterranean diet allow it to be adjusted with sustainability in mind [47]. One can easily adopt a more climatarian approach by following the Mediterranean diet and focusing on consuming seasonal, locally sourced, and sustainably harvested and processed ingredients to further minimize emissions.

#### 3.1.3. Vegan

The term “veganism” was first coined in 1944 by Donald Watson and Dorothy Morgan. Veganism has evolved from a strict form of vegetarianism, a diet that was first observed in ancient India in conjunction with the Jain principle of ahimsa, or nonviolence [48]. Today, an estimated 5% of the U.S. population is considered to be plant-based, with around 16 million adults identifying themselves as vegan or plant-based consumers [24]. Veganism has become increasingly popular and has experienced immense growth since the 2010s, which can be observed in the surge in the number of plant-based food options to choose from at restaurants and grocery store chains and of product brands specifically marketed as “vegan” to the everyday consumer. Such advancement has been announced through a rapid market expansion, as “the retail market for plant-based foods is worth $7.4 billion, up from $6.9 billion in 2020”, according to the Good Food Institute [49].

Strict plant-based diets, such as vegetarianism and veganism, are often defined by which categories of food are excluded rather than included. Both diets share the elimination of meat, whereas vegetarianism still allows for the consumption of animal products such as eggs and dairy. A true vegan avoids the use of any animal products in their diet, including meat, dairy, eggs, and animal-derived materials or ingredients (i.e., honey, rennet, and gelatin). Because of broad restrictions in the major food categories, there is an emphasis on the consumption of fruits, vegetables, grains, legumes, nuts, and seeds. Thus, veganism is typically low in fat and protein but high in carbohydrates. 

Nutritionally, “vegan diets are rich in fiber, magnesium, Fe3+, folic acid, vitamins C and E, polyunsaturated fatty acids (PUFA), carotenoids, flavonoids, other phytochemicals and antioxidants” that are often needed for the prevention of several chronic diseases [50]. Vegans that are able to eat balanced diets that fulfill their body’s essential nutritional requirements have “been observed [to have] had lower risks of obesity, hypertension, cardiovascular diseases, diabetes, arthritis, cancers (especially colon and prostate cancer) and fatal ischemic heart disease, thanks to protective substances found in fruits, vegetables, legumes, seaweed, seeds, whole grains, vegetable oils, and other plant-based foods” [51]. However, vegans are also prone to deficiencies in omega-3 fatty acids, iron, calcium, vitamin B_12_, vitamin B_2_ and B_3_, zinc, vitamin D, and iodine, which can become detrimental toward the maintenance of strong immune-system support when combined with a low intake of protein [45,50]. It is also worth mentioning that veganism has been linked to low energy levels, weight fluctuations, hormone disruption, and an increased risk of depression if a well-rounded and balanced diet is not followed [52].

There is still much academic and scientific debate about whether veganism is actually considered to be healthier than a typical omnivorous diet. A lifestyle in which one consumes low amounts of meat has been linked to an increase in life expectancy due to a reduction in the risk of developing cardiovascular disease, but the associated low protein content as well as vitamin and mineral deficiencies in the vegan diet can weaken the immune system [45,52]. Conflicting studies have yet to agree upon the nutritional soundness of veganism, but many have suggested that a well-balanced and fortified vegan meal plan can overcome many of these perceived health barriers to make the diet sustainable in the long term.

As for the environmental considerations of maintaining a vegan diet and lifestyle, an increased intake of fresh fruits and vegetables allows for a considerably lower carbon footprint compared with that of many other dietary practices. A vegan diet with low amounts of the highly manufactured and processed foods that are typical of the Standard American diet and supplemented with those that follow the likes of keto and paleo will have a lower environmental impact. However, as veganism has grown in popularity in recent years, there has been an observable increase in the number of vegan or plant-based meat alternatives that may distract from the environmental and sustainability benefits of veganism [51]. Because the alternative-meat industry continues to rapidly expand as a result of higher demand, the carbon footprint of a vegan diet may begin to trend toward that of the Standard American diet as more vegans consume higher amounts of processed plant-based meat-substitution products. However, for the purposes of this paper, a highly processed vegan diet dependent on artificial meat was not explored.

#### 3.1.4. Paleo

The paleo diet (also known as the Paleolithic diet or the Old Stone Age diet) is a style of eating that was first suggested by gastroenterologist Walter Voegtlin in the early 1970s [53] but was catapulted to mainstream popularity with the publication of Loren Cordain’s *The Paleo Diet Revised: Lose Weight and Get Healthy by Eating the Foods You Were Designed to Eat* in 2001. Today, around three percent of Americans follow this dietary practice [24].

Paleo emerged as a nutritional regimen dedicated to the re-creation of the early human diet of the Paleolithic Age (approximately 2.5 million years ago) before the influence of modern agriculture and the widespread availability of food, and even longer before complex manufacturing of processed foods became common practice. The paleo diet places a heavy emphasis on the that the human body is slow to evolve and is, therefore, gastrointestinally incompatible with the majority of foods one can buy in a grocery store today. Thus, paleo aims to limit one’s food intake to only foods that could be obtained via hunting and gathering. This means that those on paleo rely on lean meats, seeds, eggs, fruits, and vegetables as their dietary staples. As a result, the paleo diet is generally low in carbohydrates but high in protein, cholesterol, and fats. One of the main tenets of the paleo diet is the emphasis on the avoidance of processed foods, refined/artificial sugars, salt, grains, legumes, and most dairy products [54].

While scientific and academic research concerning the nutritional and health benefits of the paleo diet is still ongoing, it appears that the adoption of a Paleolithic style of eating has positive results in reducing cardiovascular disease risk factors and inducing weight loss in overweight subjects [55]. The diet also has encouraging effects, such as decreasing levels of systolic blood pressure, diastolic blood pressure, total cholesterol, and low-density lipoprotein cholesterol while increasing concentrations of high-density lipoprotein cholesterol [56]. In addition to lessening the effects of cardiovascular disease risk factors, “previous studies reported positive effects of a [paleo diet] on energy intake, body composition, [and] insulin sensitivity” [22]. Beyond the perceived health benefits, paleo has been recognized to be high in fiber, carotenes, and vitamins C and E as a result of its emphasis on the consumption of fruits and vegetables. Furthermore, the low sodium content of paleo, due to its elimination of processed foods, has often led to a decrease in blood pressure, which is another stressor in the development of cardiovascular disease [56]. However, the limitation of salt may lead to a deficiency in iodine, and the “limitation of grains and dairy [may lead to a deficiency in] vitamin D, calcium, thiamin, riboflavin, and iron” [57]. It has also been suggested that those who adopt a diet similar to that of our hunter-gatherer ancestors may experience discrete metabolic and physiological decomposition related to the improvement of lipid profiles and glucose sensitivity without inducing unhealthy weight loss [58]. Thus, it can be expected that the philosophy of the paleo diet may lead to several positive health and nutritional effects that may mask other deleterious ones.

The diet’s emphasis on lean meats and avoidance of highly processed food items may indicate a relatively moderate carbon footprint, as compared to that of the Standard American diet. Many substitutions can be made with regard to one’s selection of meats to make the paleo diet more environmentally friendly, such as lean beef, fish, or chicken as opposed to other beef and pork products that are associated with higher carbon footprints.

#### 3.1.5. Keto

The ketogenic diet, also known as keto, was originally coined in the early 20th century as a way to prevent seizures in those with epilepsy [59]. It has since been used to treat various conditions, such as brain injuries and Alzheimer’s disease [60]. The keto diet is gaining in popularity in the U.S., with about 5% of the population reporting to be on the high-fat diet [24].

Keto is characterized by the consumption of foods that are rich in fat and protein accompanied by a significant limitation of carbohydrates, such as sugars and grains. The goal is for the body to reach a state of ketosis, in which it uses fat stores instead of carbohydrates for fuel [61]. As fruits and vegetables tend to be rich in carbohydrates, they are limited to select varieties such as berries, leafy greens, broccoli, mushrooms, garlic, squash, etc. 

According to the literature, both benefits and risks to health are evident in those who adopt a keto diet. For the average person, who does not suffer from the conditions listed above, the keto diet may assist in weight loss, reducing sugar intake, improving cardiovascular-related lipid markers, microbiome healing, improving epigenetic markers, and reversing diabetes [60,62]. As the diet does not focus on caloric restriction, it has helped obese patients who had previously struggled with other diets to lose weight. However, there are also risks associated with the keto diet. There may be an excessive load on the liver and kidneys in order to process all the fat and protein from the diet, constipation due to the lack of fiber, fuzzy thinking, mood swings, an increase in LDL “bad” cholesterol, and nutritional deficiencies due to the limited range of fruits and vegetables [60]. The diet also does not restrict the consumption of the unhealthy saturated fats often found in foods such as bacon and butter. Although many individuals have achieved successful weight loss with this diet, the results vary on an individual basis and the sustainability of the diet is questionable along with its potential benefits [63,64].

The lack of restrictions on high-fat animal products and red meat indicates a high environmental impact of the keto diet. Although it is possible to alter the diet to be more climate-friendly by focusing more on white meat and plant-based foods that are high in fat, keto would inevitably make a climate-friendly diet, which is already restrictive, more difficult to maintain.

#### 3.1.6. Climatarian

The climatarian diet is a relatively new development that has a focus on eating foods that minimize one’s impact on the environment. Formulated to be flexible, there are no forbidden foods; rather, the diet emphasizes making informed decisions about the food one eats and choosing low-impact options whenever possible [6]. There is no data on the number of followers of the climatarian diet within the U.S., because the new diet has yet to become as popular as the other diets discussed. However, a Food and Health Survey by the International Food Information Council [24] found that climate-friendly diets are followed by a combined 13–15% of the population (flexitarian, plant-based, vegetarian, vegan, and pescatarian). The diet also has the potential to expand as the population becomes more conscious of the environmental impacts associated with individual behaviors such as food consumption.

The climatarian diet encourages the consumption of local, seasonal, and fresh food that requires minimal transportation, refrigeration, and processing. It also emphasizes an overall reduction in meat consumption, with a significant avoidance of ruminant meats (lamb, goat, and beef) and a moderate intake of pork, poultry, and sustainable fish. The diet is relatively flexible otherwise. It is noted that pescatarian, vegetarian, and vegan diets are all climate friendly as well, which is mainly due to the lack of consumption of red meat [6].

There is no research on the health impacts of the climatarian diet, as it is new and has yet to become popular. However, much evidence exists for an association between the reduction in red and processed meat consumption and many health benefits including reduced risks of cancer, heart disease, and stroke [65]. The consumption of high-quality plant proteins such as tofu, lentils, nuts, seeds, and vegetables can also lower the risks of cancer, heart disease, and stroke [66]. Conversely, processed foods are associated with an increase in the risk of noncommunicable disease [67]. Because the climatarian diet relies on minimally processed, locally grown foods, it may indirectly provide extra health benefits. The potential health risks of the diet have not been investigated.

The climatarian diet provides guidelines on how to eat popular foods in a more environmentally conscious manner. With a lack of restrictions on the food itself and a higher focus on its source, production, and transportation, the climatarian diet encourages its followers to focus on plant-based, whole foods while still enjoying non-red meat and animal products that are local, more sustainably sourced, and require less environmental resources. This flexibility makes the diet more accessible to some, while the high costs and inconveniences of buying local and sustainable food may render it less accessible to others.

### 3.2. Carbon Footprint Results

Table 2 contains the summarized carbon footprint values of the food items in each meal for the six comparison diets. Detailed values for each food item are found in the Supplemental Material. For the diets that customarily include ruminant meat (Standard American, paleo, and keto), we created higher and lower carbon-footprint ranges. Ruminant meats represent the highest carbon-footprint values, and any diets that include ruminant meats will automatically have higher values even though the rest of the diet may be more carbon friendly. With higher and lower footprint ranges, we were able to describe diets with ruminant meats (higher footprint) and without ruminant meats (lower footprint). The climatarian diet was also split into vegetarian and meat-eating variations to investigate the difference between its lower- and higher-impact variations.

**Figure 1:**
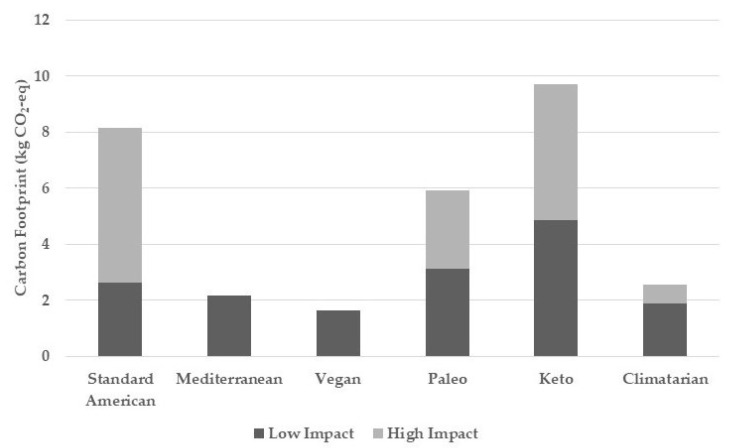
Estimated carbon-footprint values for U.S. diets, standardized to 2000 calories a day.

After curating the daily meal scenarios for each of the diet types identified above (Table 2), the carbon footprint of each food item was determined (Figure 1) using a combination of the SEL and Song et al. databases [13,14]. Looking at the meal scenarios that have the lowest environmental impacts, it was calculated that the vegan diet scenario held a footprint of 1.63 kg CO_2_-eq, the Mediterranean diet held one of 2.17 kg CO_2_-eq, and the climatarian diet ranged from 1.88 to 2.54 kg CO_2_-eq. Looking at the diets that are more dependent on ruminant meats and considered to be less environmentally sustainable, the Standard American’s footprint ranged from 2.63 to 8.14 kg CO_2_-eq, paleo’s ranged from 3.11 to 5.91 kg CO_2_-eq, and keto’s ranged from 4.85 to 9.72 kg CO_2_-eq. All of the diets’ footprints were dependent on the inclusion or exclusion of meat, particularly ruminant meat.

## 4. Discussion

The carbon-footprint estimates show that the six diets can be categorized into either higher- or lower-emission diets. The Standard American, keto, and paleo diets comprise the high-emission category (ranging from 2.63 to 9.72 kg CO_2_-eq), while the Mediterranean, vegan, and climatarian diets comprise the low-emission category (ranging from 1.63 to 2.54 kg CO_2_-eq). The primary determining factor for this observation was the regular consumption of red meat in the higher-footprint variations of the high-emission diets. When higher-impact meat, such as beef, is replaced with lower-impact meat, such as chicken or pork, there is a significant decrease in the carbon footprint of the Standard American, keto, and paleo diets. Specifically, the Standard American diet saw a reduction from 8.14 to 2.63 kg CO_2_-eq, the keto diet saw a reduction from 9.72 to 4.85 kg CO_2_-eq, and the paleo diet saw a reduction from 5.86 to 3.11 kg CO_2_-eq. To investigate the impact of meat within the climatarian diet, one meal scenario relied on lower-footprint meat-protein sources while the other scenario maintained a vegetarian diet. Removing meat protein resulted in a reduction from 2.54 kg CO_2_-eq to 1.88 kg CO_2_-eq. A similarly low estimate of 2.17 kg CO_2_-eq was found in the Mediterranean diet, which inherently emphasizes low-impact meat, such as chicken and fish, over red meat. The vegan diet, as a result of having a complete lack of animal products, was found to have the lowest carbon-footprint estimate of 1.63 kg CO_2_-eq.

These results are likely due to the carbon-dioxide emissions of beef-protein synthesis, which are estimated to be “an equivalent of 221.63 g of carbon dioxide… generated per gram of protein” [68]. Ruminant meat has an environmental impact that is 20–100 times that of plants. Non-ruminant animal products, such as milk, eggs, pork, poultry, and seafood, have impacts that are 2–25 times higher per kilocalorie of food produced than plants do. This trend held even when foods were examined per gram of protein [69]. In terms of its carbon footprint, ruminant livestock directly contributes to 38% of U.S. methane emissions, a greenhouse gas that has 28 times the global warming potential of carbon dioxide [70]. Results from the low-impact diets show that meat consumption does not need to be eliminated in order to maintain a diet with a relatively low carbon footprint. For instance, substitutions can be made with regard to one’s selection of meats to make the paleo diet more environmentally friendly, such as consuming fish, turkey, and chicken as opposed to beef and pork products, which are associated with higher carbon footprints. Therefore, a climate-friendly diet is flexible and achievable for the average person in the U.S. simply by making efforts to reduce and/or replace ruminant meat (lamb and beef) consumption.

It is important to note that diets heavy in air-freighted or processed foods will likely have higher carbon footprints. Processed foods are more likely to be found in vegan and Standard American diets, as these have no restrictions on the food category. Ultra-processed foods now constitute around 60% of the average U.S. person’s diet, although not all processed foods are created equally [71]. Minimally processed foods that are composed of whole-food ingredients tend to be healthier than the ultra-processed kind, which have mostly additives and artificial ingredients [72]. In the case of comparing processed imitation meat with red meat, the carbon footprint of the former is lower; however, the carbon footprints of meat substitutes are still higher than those of whole-food plant-protein alternatives, such as tofu and legumes, as a result of the emissions associated with processing and refrigeration [5]. The production methods of processed foods can be oriented to lower their impact on the environment, as seen in example brands such as Airly, Moonshot, etc. These climatarian products have a net negative impact on overall carbon-footprint estimates.

Our findings support the principle that diets associated with more health benefits for humans tend to also be healthier for the planet. Diets with the highest portions of red meat are often perceived as the most “unhealthy” because of their association with an increased risk of developing adverse health conditions, such as type 2 diabetes, hypertension, and many other chronic diseases related to poor diets. Our results demonstrate that the consumption of red meat is the largest contributing factor toward a high-carbon-footprint diet, as is evidenced by the drop in carbon-footprint values when meat-substitution calculations were carried out. While this is a significant revelation for combating climate change at the personal level through diet, the carbon footprint associated with a specific meat type may not be the determining factor for decreasing red-meat consumption in America. An association with more health benefits for humans may be the more important driver for dietary choice. These philosophical approaches to dietary choices may also be different depending on age group, with the elderly more likely to value health benefits while younger age groups may place more importance on environmental impact. There are also potential barriers against replacing meat with plant-based protein sources, such as “the sensory experience of eating meat, the taste and subjective concerns about the risk of protein deficiency” [73].

Vegan, Mediterranean, and climatarian diets, in addition to having health benefits, also fulfill the low-impact criteria, meaning that planet health is inherently prioritized in these diets. One might further argue that the keto and paleo diets—both of which have shown efficacy in promoting weight loss and lowering heart-disease risk—can also reduce their carbon footprints by decreasing their reliance on red-meat consumption. The EAT-Lancet Commission report noted that “dietary changes from current diets towards healthy (flexitarian) diets are likely to result in significant health benefits that include averting approximately 7.4 to 10.8 million premature deaths per year, a reduction of between 18% to 28%” [5]. Even though as many as 16 million Americans claim to lead a plant-based lifestyle, health providers such as Kaiser Permanente have noted that “despite a continuously growing body of evidence and the meticulous work of renowned experts in this field worldwide, the latest findings in this area have not found their way into US national dietetic guidelines” [74]. The U.S. Dietary Guidelines are updated every five years and rely on meta-analyses of previously published studies, yet they have not yet included plant-based diets nor have they incorporated environmental impacts. The increasing popularity and expansion of largely plant-based or flexitarian diets (such as the climatarian and Mediterranean diets) points toward a movement to lower the carbon footprint associated with food consumption.

Many U.S. residents adopt plant-based or low-meat diets with the goal of weight loss. With the obesity epidemic affecting 41.9% of U.S. adults, the inherently reduced caloric density of low-impact category diets allows for substantial progress in the loss of body fat without extensive caloric restriction or the integration of an extensive exercise regime, instead focusing on simple food swaps in high-impact categories such as protein sources [75]. For example, a meta-analysis conducted in 2019 found that “participants with a weight loss >7% of initial body weight ate more portions of fruits and vegetables/day compared to those who achieved less weight loss (<2%),” meaning that an increased intake of fresh produce in place of meat and processed carbohydrates was positively associated with weight loss [76]. Because of the low carbon footprint of fresh produce, this is further evidence that weight loss and environmental considerations appear to coincide with one another. The motivation to adopt diets that have the highest likelihood of weight loss could be further bolstered by the idea of eating to benefit not only one’s personal health but also the health of the environment.

The limitations of this study must also be accounted for and discussed accordingly. First, only one component of the environmental impact of food items was evaluated; other factors, such as the water footprint, were not included because of limited data. Second, a single day of meals does not capture the full impact potential of the diets. Each meal plan scenario is intended to be a succinct snapshot of a single day of eating; a different menu, even while still adhering to the dietary restrictions, could potentially have a higher or lower carbon footprint. Third, meals are likely to underestimate the total calories that most Americans eat. The FDA emphasizes that their 2000-calorie daily estimate is far lower than the observed 3800 calories consumed by the average American [77]. Thus, it can be assumed that the calculated carbon footprints are a vast underestimate of the ultimate environmental impact produced by food consumption.

Finally, the emissions estimates are assuredly lower than actual emissions because they do not include emissions associated with the post-farm-gate components of the life cycle of each food item. The SEL and Song et al. databases provide the most accurate and current information concerning the carbon footprints of a wide range of food items, but neither dataset accounts for transportation in their provided carbon-footprint values [13,14]. The two databases instead focused on the carbon footprints of activities before leaving the farm gate, meaning that the environmental burdens associated with procedures such as packaging, market transportation, warehousing, and refrigeration or the energy required for food preparation within the home of the consumer was not included in their carbon-footprint calculations. While it may seem significant that transportation was not included in their carbon-footprint calculations, transportation has a minimal impact on the environmental burden associated with food consumption unless the food item is transported via air [78]. In most cases, transportation only accounts for less than 10% of the total emissions associated with a food product [5]. Because of this, the carbon-footprint estimates of eating local food items would likely only have been marginally lower than those of food distributed from regional centers. This is particularly relevant to the climatarian diet, as one of its main principles is to defer to local food sources, such as farmers markets and food stands, that may have food that has traveled less than 100 miles from farm to market. While this alleviates concerns about the lack of data regarding post-farm-gate activities, it may hold true that other climatarian principles, such as reducing animal-product consumption (especially ruminant meat, such as lamb and beef) or eating seasonal foods (decreasing the demand for items not in season in one’s specific location, thereby decreasing the need for food to travel long distances from far away farms where the particular food item is in season), may have a larger effect on reducing the overall carbon footprint [79]. Food waste is another post-farm-gate component that contributes to excess greenhouse gas emissions from food. Food waste accounts for about 34% of GHG (greenhouse gas) emissions related to an individual’s food-related resource consumption in the United States. The EAT-Lancet Commission found that food waste must be halved in order to be in line with global sustainable development goals and stay within a safe operating space [5].

It would be beneficial for more concrete and freely accessible data to be gathered and formatted to better inform studies similar to this one. To the best of our knowledge, the U.S. lacks the same free and accessible scholarly databases of the carbon footprints of a variety of individual food items that the European Union has. There are even fewer datasets available on water footprints and other measures of environmental impact at a refined level for food consumption. The ongoing evolution of science should emphasize affordable and accessible means of performing life-cycle assessments for all potential human behaviors to more efficiently inform behavioral changes in favor of human and planetary health, particularly in light of an increasingly evident climate change. In expanding upon the research presented in this study, it would be worthwhile to investigate the extensive (and almost infinite) variation in diets by altering or completely redesigning the meal-plan scenarios introduced here. For instance, one could look at higher caloric intakes more akin to the calorie-excessive diet of the average American. Lowering caloric intake would also lower carbon footprint because less food would be consumed. Another option could be factoring name-brand products with their respective carbon-footprint values, as different production or manufacturing processes could significantly alter the carbon footprint of a meal. Finally, calculating a full week of menus may expose how certain foods have disproportionate impacts. Another suggestion for ongoing evaluation could be to analyze the environmental impacts of different age groups, sexes, and levels of physical activity, as the caloric needs for each group are often drastically different. As one can see, the potential to expand upon the work at hand is extensive, and, therefore, it is easy to see why the limitations of this study were necessary and numerous.

## 5. Conclusions

The unique intersection of planetary and human health in diets presents a great opportunity. A diet is a direct vehicle for sustainability at the individual level. The environmental impact of human behavior is increasingly weighing on the minds of many as climate change continues to unfold and worsen. As this study illuminates, diets such as Standard American, paleo, and keto have higher carbon footprints compared with diets such as Mediterranean, vegan, and climatarian. Unsurprisingly, the consumption of animal products, especially ruminant meat, was the most influential factor leading to this observation.

The climatarian diet has the potential to be further popularized with the help of stakeholders, ambassadors, and influencers within corporate and social media landscapes. Corporate stakeholder campaigns, such as Imperfect Foods [80], Panera Bread’s “Cool Food Meals” [81], Weight Watchers’ low-impact meal options [82], and Lifesum’s option for selecting a climatarian diet [83], are all examples of the promotion of a more climate-friendly diet. Many professional chefs have taken on climatarian cooking, centering around a lifestyle that values local, fresh, whole, and seasonal ingredients. In an interview with CNN [84], a Los Angeles-based chef, named Camilla Marcus, emphasized looking to the past for inspiration on climatarianism, when zero-waste and sustainable eating was the only option in most cases. Another chef, Rasheeda McCallum, works with the Black Chef Movement to collaborate with community gardens to make local and fresh food accessible to all [84]. As paleo and keto diets, which tend to emphasize high-impact animal products, continue to increase in popularity in the U.S., it is important that their environmental implications and other flexible, more sustainable options, such as the climatarian and Mediterranean diets, are brought to the awareness of the general public at the same time.

This review focused mainly on the carbon footprints and health impacts of select diets, along with potential solutions at the individual level. However, in order to achieve a truly sustainable food system, the problem must be tackled from multiple dimensions. In addition to the role of the individual consumer, there are important roles for farmers, corporations, and policy makers in working toward mitigating climate change through healthy and sustainable diets. A future of food consumption that benefits both humans and the environment should be flexible and involve an emphasis on whole, plant-based foods combined with a reduced consumption of meat and processed food. These behavioral changes can be supported by increasing the availability and accessibility of detailed food-life-cycle assessments.

## Figures and Tables

**Table 2:** Summary of Carbon Footprint Results.

Diet	Breakfast (kg CO_2_-eq)	Snacks (kg CO_2_-eq)	Lunch (kg CO_2_-eq)	Dinner (kg CO_2_-eq)	Total (kg CO_2_-eq)
Standard American	0.51	0.61	0.64	Lower Footprint: 0.87 Higher Footprint: 6.38	Lower Footprint: 2.63 Higher Footprint: 8.14
Mediterranean	0.61	0.47	0.50	0.59	2.17
Vegan	0.39	0.20	0.37	0.67	1.63
Paleo	1.49	0.22	Lower Footprint: 0.61 ^1^ Higher Footprint: 3.41 ^2^	0.78	Lower Footprint: 3.11 Higher Footprint: 5.91
Keto	1.52	0.56	1.00	Lower Footprint: 1.77 Higher Footprint: 6.64	Lower Footprint: 4.85 Higher Footprint: 9.72
Climatarian	0.65	0.30	Vegetarian: 0.29 Meat-Eating: 0.83	Vegetarian: 0.64 Meat-Eating: 0.76	Vegetarian: 1.88 Meat-Eating: 2.54

^1^ Lower footprint refers to using white meat (chicken, pork) for protein, ^2^ Higher footprint refers to using red meat (beef, lamb) for protein.

## Data Availability

Not applicable.

## Supplementary Materials

The following supporting information can be downloaded at: https://www.mdpi.com/article/10.3390/nu15030692/s1, Tables S1–S6: Full Detail Meal Scenarios, Nutrition and Carbon Footprint Totals for the Standard American, Mediterranean, vegan, paleo, keto, and climatarian diets. Refs. [6,13,14,15,18,19,20,21,22,23] are cited in Supplementary Material.
