# Supplementary material for: Modern Diets and the Health of Our Planet: An Investigation into the Environmental Impacts of Food Choices
Source: Nutrients. 2023 Jan 30;15(3):692. doi: 10.3390/nu15030692 (PMC9919910; doi:10.3390/nu15030692)
Supplement: Supplementary file 1 [file nutrients-15-00692-s001.zip › nutrients-2168430-supplementary.pdf]

Supplementary Material: Modern Diets and the Health of Our Planet: An Investigation into the Environmental Impact of Food Choices

Full Detail Meal Scenarios, Nutrition Totals, and Carbon Footprint Totals

Table of Contents

S1: Standard American
S2: Mediterranean
S3: Vegan
S4: Paleo
S5: Keto
S6: Climatarian

Notes

- Nutrition category percentages and totals may not exactly add up to 100% or total estimate due to rounding.
- “CHO” column totals include both fiber and carbohydrates.

Legend

*Avocado is from Central America carbon footprint estimate.
†Carbon Footprint Calculation for Airly Crackers: -21 g CO2 / 213 g box = -2.85915 g CO2 / 29 g serving
**Items purchased from local farmer’s market

Table S1.1.1. Standard American Higher Impact Meal Scenario Meal Plan Overview

Meal [3,4]	Food Item	Quantity	Kcal	CHO	FAT	PRO	FIBER	Food Item from Database: Petersson [1], Song [2]	CF (kg CO2eq/kg food)	Food Weight (g)	Carbon Footprint (kg CO2eq)
**Breakfast**	Yogurt - vanilla low fat - Greek (Dannon)	1 container - 6 oz	140	18	4	14	0	Yogurt White [1]	1.5	170.1	0.26
	Whole wheat toast	1 slice	85	5	1	4	3	Bread [2]	1	30	0.03
	Butter	1 tbsp	102	0	12	0.12	0	Butter [1]	7.3	14.1	0.10
	Banana	1 medium	105	26.9	0.4	1.29	3.07	Fruits [2]	1	118	0.12
**Snack**	Cheddar cheese	2 slice	140	0.4	7.4	4	0	Cheddar [1]	11.207	42	0.47
	Whole wheat crackers - Triscuit	6 crackers	120	20	3.5	3	3	Bakery products (sweet and salty)	2	28	0.06
**Lunch**	Leafy salad greens	2 cups	20	3	0.26	1	1.8	Spinaches [2]	0.2	100	0.02
	Tomato	1 medium	22	4	0.4	0.7	2	Tomato [1]	0.475	70	0.03
	Chopped onions	50 g	22	5	0	0.5	2	Onion [2]	0.5	50	0.03
	Turkey	4 slices	84	0	3.5	12	0	Turkey bone free meat [1]	2.807	168	0.47
	Salad dressing - Italian (Ken's Steak	1 tbsp	75	0	8.5	0	0	Sauces, seasonings, and condiments [2]	4	15.5	0.06

	House)										
	Whole wheat bread	1 slice	85	5	1	4	3	Bread [2]	1	30	0.03
**Snack**	Apple	1	62	18	0	0.2	6	Apple [1]	0.358	154	0.06
	Peanut butter	1 tbsp	100	4	8	4	1.3	Peanut butter & peanut paste [1]	1.75	15	0.03
**Dinner**	Lean beef	8 oz	440	0	18	65	0	Beef Bone Free Meat [1]	26.821	226.8	6.08
	Baked potato with skin	1 medium	161	41	0	5	3.8	Potato [1]	0.32	173	0.06
	Asparagus	0.5 cup	28	8	0	2	3	Asparagus [1]	0.915	44	0.04
	Butter	2 tbsp	204	0	24	0.24	0	Butter [1]	7.3	28.2	0.21

Table S1.1.2. Standard American Higher Impact Meal Scenario Nutrition Overview

**Nutrition [3]**	**Kcal**	**CHO**	**FAT**	**PRO**	**FIBER (CHO)**
**Totals**	1995	158.3	91.96	121.05	31.97
Calories		633.2	827.64	484.2	127.88
Percentages		31.74%	41.49%	24.27%	6.41%

Table S1.1.3. Standard American Higher Impact Meal Scenario Carbon Footprint Totals

**Carbon Footprint**	**Kg CO_2eq**
Breakfast	0.51

Lunch	0.64
Snacks	0.61
Dinner	6.38
**Total**	**8.14**

Table S1.2.1. Standard American Lower Impact Meal Scenario Meal Plan Overview

**Meal [3,4]**	**Food Item**	**Quantity**	**Kcal**	**CHO**	**FAT**	**PRO**	**FIBER**	**Food Item from Database: Petersson [1], Song [2]**	**CF (kg CO2eq/kg food)**	**Food Weight (g)**	**Carbon Footprint (kg CO2eq)**
**Breakfast**	Yogurt - vanilla low fat - Greek (Dannon)	1 container - 6 oz	140	18	4	14	0	Yogurt White [1]	1.5	170.1	0.26
	Whole wheat toast	1 slice	85	5	1	4	3	Bread [2]	1	30	0.03
	Butter	1 tbsp	102	0	12	0.12	0	Butter [1]	7.3	14.1	0.10
	Banana	1 medium	105	26.9	0.4	1.29	3.07	Fruits [2]	1	118	0.12
**Snack**	Cheddar cheese	2 slice	140	0.8	14.8	8	0	Cheddar [1]	11.207	42	0.47
	Whole wheat crackers - Triscuit	6 crackers	120	20	3.5	3	3	Bakery products (sweet and salty)	2	28	0.06
**Lunch**	Leafy salad greens	2 cups	20	3	0.26	1	1.8	Spinaches [2]	0.2	100	0.02

	Tomato	1 medium	22	4	0.4	0.7	2	Tomato [1]	0.475	70	0.03
	Chopped onions	50 g	22	5	0	0.5	2	Onion [2]	0.5	50	0.03
	Turkey	4 slices	84	0	3.5	12	0	Turkey bone free meat [1]	2.807	168	0.47
	Salad dressing - Italian (Ken's Stakehouse)	1 tbsp	75	0	8.5	0	0	Sauces, seasonings, and condiments [2]	4	15.5	0.06
	Whole wheat bread	1 slice	85	5	1	4	3	Bread [2]	1	30	0.03
**Snack**	Apple	1 medium	62	18	0	0.2	6	Apple [1]	0.358	154	0.06
	Peanut butter	1 tbsp	100	4	8	4	1.3	Peanut butter & peanut paste [1]	1.75	15	0.03
**Dinner**	Chicken breast barbequed with skin	1 breast	275	0	13.7	108	0	Chicken Bone Free Meat [1]	3.277	174	0.57
	Baked potato with skin	1 medium	161	41	0	5	3.8	Potato [1]	0.32	173	0.06
	Asparagus	0.5 cup	28	8	0	2	3	Asparagus [1]	0.915	44	0.04
	Butter	2 tbsp	204	0	24	0.24	0	Butter [1]	7.3	28.2	0.21

Table S1.2.2. Standard American Lower Impact Meal Scenario Nutrition Overview

**Nutrition [3]**	**Kcal**	**CHO**	**FAT**	**PRO**	**FIBER (CHO)**
**Totals**	1830	158.7	95.06	168.05	31.97

Calories		330.4	855.54	672.2	98.4
Percentages		18.05%	46.75%	36.73%	5.38%

Table S1.2.3. Standard American Lower Impact Meal Scenario Carbon Footprint Totals

Carbon Footprint	Kg CO_2eq
Breakfast	0.51
Lunch	0.64
Snacks	0.61
Dinner	0.87
**Total**	**2.60**

Table S2.1. Mediterranean Meal Plan Overview

Meal [3,5]	Item	Quantity	Kcal	CHO	FAT	PRO	FIBER	Food Item from Database: Petersson [1], Song [2]	CF (kg CO2eq/ kg food)	Food Weight (g)	Carbon Footprint (kg CO2eq)
**Breakfast**	Strawberries	1/2 cup	40	13.5	0	0	1.5	Blueberries [1]	0.86	190	0.16
	Blueberries	1/2 cup	40	13.5	0	0	1.5	Strawberries [1]	0.37	100	0.04
	Non-fat greek yogurt	1/2 cup	100	0	0	15	0	White Yogurt [1]	1.5	123	0.18
	Eggs	1 boiled	140	0	5	14	0	Eggs [1]	4.46	50	0.22

**Snack**	Walnuts	2 oz	327	7	32	7.5	3.4	Walnut [1]	1.48	56	0.08
	Whole wheat crackers - Triscuit	6 crackers	120	20	3.5	3	3	Bakery products [2]	2	28	0.06
	Mozzarella cheese	1 oz	90	0	7	7	0	Mozzarella [1]	7.79	28	0.22
**Lunch**	Whole Wheat bread	2 slices	200	32	4	8	4	Wheat Grains [1]	0.56	70	0.04
	Turkey breast lunch meat	2 oz	60	1	2	8	0	Turkey Bone Free Meat [1]	3.83	57	0.22
	Mayonnaise	1 tbsp	90	0	10	0	0	Sauces, Seasonings, Condiments [2]	4	14	0.06
	Spinach	2 cups	17	1.6	0	1.7	1	Spinaches [2]	0.2	60	0.01
	Tomato	1 medium	22	4	0.4	0.7	2	Tomato [1]	0.47	70	0.03
	Cucumber	1/4 cup	6	1.5	0	0	0.2	Cucumber [2]	3	40	0.12
	Olive oil	1 tbsp	64	11	5	0	0	Olive Oil [2]	2	13	0.03
**Snack**	Carrot sticks	1 cup	50	12	0	1	3	Carrot [2]	0.3	100	0.03
	Apple	1 apple	95	25	0	4	4	Apple [1]	0.36	182	0.07
	Peanut butter	1 tbsp	100	4	8	4	1.3	Peanut Butter [1]	1.15	15	0.02
**Dinner**	Salmon	4.2 oz	281	0	12.6	39.2	0	Salmon [1]	3.47	85	0.29
	Whole wheat pasta	1/2 cup	92	19	0	3.4	3.5	Pasta [1]	1.656	50	0.08
	Spring mix	1 cup	20	10	0	0	2.5	Lettuce [1]	0.33	50	0.02

	Cucumber	1/4 cup	6	1.5	0	0	0.2	Cucumber [2]	3	40	0.12
	Carrots	1/4 cup	12.5	3	0	0.25	0.75	Carrot [2]	0.3	40	0.01
	Olive oil	1 tbsp	64	11	5	0	0	Olive oil [2]	2	13	0.03
	Broccoli	1 cup	25	6	0	2	3	Broccoli [1]	0.5	71	0.04

Table S2.2. Mediterranean Nutrition Overview

**Nutrition [3]**	**Kcal**	**CHO**	**FAT**	**PRO**	**FIBER(CHO)**
**Totals**	2036	190.54	94.5	117	32
Calories		762.16	850.5	467	7
Percentages		37%	42%	23%	17%

Table S2.3. Mediterranean Carbon Footprint Totals

**Carbon Footprint**	**Kg CO_2eq**
Breakfast	0.61
Lunch	0.47
Snacks	0.50
Dinner	0.59
**Total**	**2.13**

Table S3.1. Vegan Meal Plan Overview

Meal [3,6]	Food Item	Quantity	Kcal	CHO	FAT	PRO	FIBER	Food Item from Database: Petersson [1], Song [2]	CF (kg CO2eq/kg food)	Food Weight (g)	Carbon Footprint (kg CO2eq)
**Breakfast**	Avocado	1 medium	233	12	22	2	10	*Avocado [1]	1.38	150	0.21
	Pinto beans - canned, drained	1/2 cup	110	21	1.25	7	5	Bean (pinto dried) [1]	0.73	130	0.09
	Olive oil	1 tbsp	120	0	13.5	0	0	Olive oil [2]	2	13.3	0.03
	Whole grain bread	1 slice	85	5	1	4	3	Bread [2]	1	30	0.03
	Tomato	1 medium	22	4.78	0.25	1	1.48	Tomato [1]	0.48	70	0.03
**Snack**	Hummus - Sabra Classic	2 tbsp	70	4	5	2	1	Sauces, seasonings and condiments [2]	4	28	0.11
	Carrots	1/2 cup	26	6	0	0.6	2	Carrots [2]	0.3	120	0.04
**Lunch**	Spinach	1 cup	35	4.42	1.5	3	4.1	Spinaches [2]	0.2	100	0.02
	Strawberries	1 cup	48.6	11.7	0.4	1	3.04	Strawberry [1]	0.43	152	0.0
	Onions	50 g	22	5	0	0.5	2	Onion [2]	0.5	50	0.025
	Almonds, roasted	1/2 cup	320	12	20	12	6	Almond [1]	2.25	60	0.1347
	Salad dressing - strawberry vinaigrette - Hanleys	2 tbsp	30	5	0.5	0	0	Sauces, seasonings, and condiments [2]	4	32	0.128

**Snack**	Apple	1	31	9	0	0.1	3	Apple [1]	0.36	77	0.027566
	Peanut butter	1 tbsp	94	3.5	8	3.7	1.3	Peanut Butter & Peanut Paste [1]	1.75	15	0.02625
**Dinner**	Tofu, 1 firm	150g	127.5	1.5	6.3	16.35	1.35	Tofu [1]	1.072	150	0.1608
	Spinach	2 cups	126	24.6	2.66	19	15	Spinaches [2]	0.2	250	0.05
	White rice	3/4 cup	539	119.7	1.95	10.42	0.75	Rice [1]	2.63	150	0.3945
	Soy sauce- Kikkoman	1 tbsp	10	1	0	2	0	Sauces, seasonings, and condiments [2]	4	15	0.06

Table S3.2. Vegan Nutrition Overview

**Nutrition [3]**	**Kcal**	**CHO**	**FAT**	**PRO**	**FIBER (CHO)**
**Totals**	1983.1	250.2	82.32	84.67	59.02
Calories		1000.8	740.88	338.68	236.08
Percentages		48.84%	37.36%	17.08%	11.90%

Table S3.3. Vegan Carbon Footprint Totals

**Carbon Footprint**	**Kg CO_2eq**
Breakfast	0.39
Lunch	0.37
Snacks	0.20
Dinner	0.67

Total	1.63

Table S4.1.1. Paleo Higher Impact Meat Scenario Meal Plan Overview

Meal [3,7-8]	Food Item	Quantity	Kcal	CHO	FAT	PRO	FIBER	Food Item from Database: Petersson [1], Song [2]	CF (kg CO2eq/kg food)	Food Weight (g)	Carbon Footprint (kg CO2eq)
**Breakfast**	Sweet potatoes - baked w/ skin	2	180	34.8	0.3	4	6.6	Potato [1]	0.32	226	0.07
	Eggs	4	286	2	19	25	0	Eggs [1]	4.46	240	1.0
	Prosciutto - Applegate	2 slices	120	0	7	14	0	Pork Bone Free Meat [1]	6.104	56	0.34
	Olive oil	2 tsp	80	0	4.5	0	0	Olive Oil [2]	2	4.44	0.009
**Snack**	Almonds	1/4 cup	225	5.48	18.02	6.88	3.7	Almond [1]	2.61	30	0.08
**Lunch**	Leafy salad greens	1 cup	10	2	0.13	0.5	1	Spinaches [2]	0.2	50	0.01
	New York strip steak (grass fed)	115 g	145	0	5.32	22.7	0	Beef Bone Free Meat [1]	26.821	115	3.08
	Avocado	1 medium	233	12	22	2	10	*Avocado [1]	1.38	150	0.21

	Chimichurri sauce	28 g	44	2	3.5	0	0	Sauces, seasonings, and condiments [2]	4	28	0.11
**Snack**	Sliced apple	1 medium	96	18	0.25	0.2	4	Apple [1]	0.358	154	0.06
	Unsweetened almond butter	2 tbsp	100	6.04	17	6	3.3	Almond Paste [1]	2.98	30	0.09
**Dinner**	Roasted snapper fish	1 filet	218	0	2.92	44.7	0	Salmon [1]	3.465	170	0.59
	Sauteed kale greens	1/2 cup	80	10	1	4	4	Spinaches [2]	0.2	226.796	0.045
	Olive oil	2 tbsp	240	0	13.5	0	0	Olive oil [2]	2	26.6	0.05
	Garlic	2 cloves	10	0.8	0	0.4	0.1	Garlic [2]	0.5	10	0.005
	Cauliflower rice	85 g	20	4	0	2	2	Cauliflower [2]	1	85	0.085

Table S4.1.2. Paleo Higher Impact Meat Scenario Nutrition Overview

**Nutrition [3]**	**Kcal**	**CHO**	**FAT**	**PRO**	**FIBER (CHO)**
**Totals**	2087	97.12	114.45	132.28	34.7
Calories		388.46	1030	529.5	138.8
Percentages		18.61%	49.35%	25.37%	6.65%

Table S4.1.2. Paleo Higher Impact Meat Scenario Carbon Footprint Totals

**Carbon Footprint**	**Kg CO2eq**
Breakfast	1.49
Lunch	3.41

Snacks	0.22
Dinner	0.78
**Total**	**5.91**

Table S4.2.1. Paleo Lower Impact Meat Scenario Meal Plan Overview

Meal [3,7-8]	Food Item	Quantity	Kcal	CHO	FAT	PRO	FIBER	Food Item from Database: Petersson [1], Song [2]	CF (kg CO2eq/kg food)	Food Weight (g)	Carbon Footprint (kg CO2eq)
**Breakfast**	Sweet potatoes - baked w/ skin	2	180	34.8	0.3	4	6.6	Potato [1]	0.32	226	0.07
	Eggs	4	286	2	19	25	0	Eggs [1]	4.46	240	1.07
	Prosciutto - Applegate	2 slices	120	0	7	14	0	Pork Bone Free Meat [1]	6.104	56	0.34
	Olive oil	2 tsp	80	0	4.5	0	0	Olive Oil [2]	2	4.44	0.009
**Snack**	Almonds	1/4 cup	225	5.48	18.02	6.88	3.7	Almond [1]	2.61	30	0.08
**Lunch**	Leafy salad greens	1 cup	10	2	0.13	0.5	1	Spinaches [2]	0.2	50	0.01
	Chicken breast - free range w/ skin barbequed	1/2 breast	137.5	0	13.7	108	0	Chicken Bone Free Meat [1]	3.277	87	0.29
	Avocado	1	233	12	22	2	10	*Avocado [1]	1.38	150	0.21

		medium								
	Chimichurri sauce	28 g	44	2	3.5	0	0	Sauces, seasonings, and condiments [2]	4	28	0.11
**Snack**	Sliced apple	1 medium	96	18	0.25	0.2	4	Apple [1]	0.358	154	0.055
	Unsweetened almond butter	2 tbsp	100	6.04	17	6	3.3	Almond Paste [1]	2.98	30	0.09
**Dinner**	Roasted snapper fish	1 filet	218	0	2.92	44.7	0	Salmon [1]	3.465	170	0.59
	Sauteed kale greens	1/2 cup	80	10	1	4	4	Spinaches [2]	0.2	226.796	0.045
	Olive oil	2 tbsp	240	0	13.5	0	0	Olive oil [2]	2	26.6	0.05
	Garlic - 2 cloves	2 cloves	10	0.8	0	0.4	0.1	Garlic [2]	0.5	10	0.005
	Cauliflower rice	85 g	20	4	0	2	2	Cauliflower [2]	1	85	0.085

Table S4.2.2. Paleo Lower Impact Meat Scenario Nutrition Overview

**Nutrition [3]**	**Kcal**	**CHO**	**FAT**	**PRO**	**FIBER (CHO)**
**Totals**	2079.5	97.12	122.83	217.68	34.7
Calories		388.46	1105.43	870.7	138.8
Percentages		18.68	53.16%	41.87%	6.67%

Table S4.2.3. Paleo Lower Impact Meat Scenario Carbon Footprint Totals

**Carbon Footprint**	**Kg CO2eq**
Breakfast	1.49

Lunch	0.61
Snacks	0.22
Dinner	0.78
**Total**	**3.11**

Table S5.1.1. Keto Higher Impact Meat Scenario Meal Plan Overview

**Meal [3,9]**	**Item**	**Quantity**	**Kcal**	**CHO**	**FAT**	**PRO**	**FIBER**	**Food Item from Database: Petersson [1], Song [2]**	**CF (kg CO2eq/ kg food)**	**Food Weight (g)**	**Carbon Footprint (kg CO2eq)**
**Breakfast**	Breakfast sausage - Farmer John	3 links	210	0	18	9	0	Sausages [2]	10	65	0.65
	Scrambled eggs	2 eggs	148	1	10	12	0	Eggs [1]	4.46	100	0.45
	Butter	1 tbsp	102	0	12	0.12	0	Butter [1]	7.3	14.1	0.10
	Cheddar cheese	1 slice	70	0.4	7.4	4	0	Cheddar [1]	11.207	21	0.235
	Bell pepper	1/4 pepper	6	1.25	0	0.2	0.2	Pepper [2]	3	30	0.09
**Snack**	Bell pepper	3/4 pepper	16	3.75	0	0.6	0.6	Pepper [2]	3	90	0.27
	Ranch dressing - Hidden	2 tbsp	130	1	13	0	0	Sauces,	4	60	0.24

	Valley Keto Friendly							seasonings, condiments [2]			
**Lunch**	Leafy salad greens	1 cup	10	1.5	0.13	0.5	0.9	Lettuce [1]	0.325	50	0.016
	Tomato	1 medium	22	4	0.4	0.7	2	Tomato [1]	0.475	70	0.033
	Celery	2 stalks	22	5	0	0.5	2	Celery [2]	1	50	0.05
	Tuna packed in water	4 oz	85	0	3.5	12	0	Fish [2]	4	113	0.45
	Avocado	1	227	12	22	2	10	*Avocado [1]	1.38	150	0.21
	Mayonnaise - Best Foods	2 tbsp	200	0	11	0	0	Sauces, seasonings, condiments [2]	4	60	0.24
**Snack**	Celery	1 stalk	62	18	0	0.2	6	Celery [2]	1	25	0.025
	Cream cheese	2 tbsp	70	1.2	6.8	1.2	0	(Fresh Cheese) Cream [1]	2.17	10	0.02
**Dinner**	Lean beef	8 oz	448	0	18	65	0	Beef bone free meat [1]	26.821	226.8	6.08
	Cauliflower - steamed	1/2 head	73.5	15	1	1	6	Cauliflower [2]	1	300	0.3
	Cheddar cheese	1 slice	70	0.4	7.4	4	0	Cheddar [1]	11.207	21	0.235
	Olive oil	1 tbsp	120	0	7	0	0	Olive oil [2]	2	13.3	0.027
	Spring mix	1 cup	20	10	0	0	2.5	Lettuce [1]	0.325	50	0.016
	Cucumber	1/4 cup	6	1.5	0	0	0.2	Cucumber [2]	3	40	0.12

	Carrots	1/4 cup	12.5	3	0	0.25	0.75	Carrot [2]	0.3	40	0.01
	Olive oil	1 tbsp	64	11	5	0	0	Olive oil [2]	2	13	0.03
	Broccoli	1 cup	25	6	0	2	3	Broccoli [1]	0.5	71	0.0355

Table S5.1.2. Keto Higher Impact Meat Scenario Nutrition Overview

**Nutrition [3]**	**Kcal**	**CHO**	**FAT**	**PRO**	**FIBER(CHO)**
**Totals**	2092	64.5	137.63	113.02	27.7
Calories		258	1238.67	452.08	110.8
Percentages		12.34%	59.22%	21.62%	5.30%

Table S5.1.3. Keto Higher Impact Meat Scenario Carbon Footprint Totals

**Carbon Footprint**	**Kg CO_2eq**
Breakfast	1.52
Lunch	1.00
Snacks	0.98
Dinner	6.64
**Total**	**9.72**

Table S5.2.1. Keto Lower Impact Meat Scenario Meal Plan Overview

**Meal [3,9]**	**Item**	**Quantity**	**Kcal**	**CHO**	**FAT**	**PRO**	**FIBER**	**Food Item from Database: Petersson [1],**	**CF (kg CO2eq/ kg food)**	**Food Weight (g)**	**Carbon Footprint (kg**

								**Song [2]**			**CO2eq)**
**Breakfast**	Breakfast sausage - Farmer John	3 links	210	0	18	9	0	Sausages [2]	10	65	0.65
	Scrambled eggs	2 eggs	148	1	10	12	0	Eggs [1]	4.46	100	0.45
	Butter	1 tbsp	102	0	12	0.12	0	Butter [1]	7.3	14.1	0.10
	Cheddar cheese	1 slice	70	0.4	7.4	4	0	Cheddar [1]	11.207	21	0.235
	Bell pepper	1/4 pepper	6	1.25	0	0.2	0.2	Pepper [2]	3	30	0.09
**Snack**	Bell pepper	3/4 pepper	16	3.75	0	0.6	0.6	Pepper [2]	3	90	0.27
	Ranch dressing - Hidden Valley Keto Friendly	2 tbsp	130	1	13	0	0	Sauces, seasonings, condiments [2]	4	60	0.24
**Lunch**	Leafy salad greens	1 cup	10	1.5	0.13	0.5	0.9	Lettuce [1]	0.325	50	0.016
	Tomato	1 medium	22	4	0.4	0.7	2	Tomato [1]	0.475	70	0.033
	Celery	2 stalks	22	5	0	0.5	2	Celery [2]	1	50	0.05
	Tuna packed in water	4 oz	85	0	3.5	12	0	Fish [2]	4	113	0.45
	Avocado	1 medium	227	12	22	2	10	*Avocado [1]	1.38	150	0.21
	Mayonnaise - Best Foods	2 tbsp	200	0	11	0	0	Sauces, seasonings, condiments [2]	4	60	0.24

**Snack**	Celery	1 stalk	11	2.5	0	0.25	1	Celery [2]	1	25	0.025
	Cream cheese	2 tbsp	70	1.2	6.8	1.2	0	(Fresh Cheese) Cream [1]	2.17	10	0.022
**Dinner**	Pork chop	8 oz	374	0	21	65	0	Pork Meat [1]	4.9705	227	1.13
	Cauliflower - steamed	1/2 head	73.5	15	1	1	6	Cauliflower [2]	1	300	0.3
	Cheddar cheese	1 slice	70	0.4	7.4	4	0	Cheddar [1]	11.207	21	0.235
	Butter	1 tbsp	102	0	12	0.12	0	Butter [1]	7.3	14.1	0.10

Table S5.2.2. Keto Lower Impact Meat Scenario Nutrition Overview

**Nutrition [3]**	**Kcal**	**CHO**	**FAT**	**PRO**	**FIBER(CHO)**
**Totals**	1949	49	145.63	113.19	22.7
Calories		196	1310.67	452.76	90.8
Percentages		6.90%	67.27%	23.24%	4.66%

Table S5.2.3. Keto Lower Impact Meat Scenario Carbon Footprint Totals

**Carbon Footprint**	**Kg CO_2eq**
Breakfast	1.52
Lunch	1.00
Snacks	0.98
Dinner	1.77
**Total**	**4.85**

Table S6.1.1. Climatarian Meat (Higher Impact) Scenario Meal Plan Overview

Meal [3,10]	Item	Quantity	Kcal	CHO	FAT	PRO	FIBER	Food Item from Database: Petersson [1], Song [2]	CF (kg CO2eq/ kg food)	Food Weight (g)	Carbon Footprint (kg CO2eq)
**Breakfast**	*Eggs	2	233	12	22	2	10	Eggs [1]	4.46	100	0.45
	*Green onion	1/2 cup	11	2	0	0.34	0.6	Onion [2]	0.5	26	0.01
	Olive Oil - in-state made	1 tbsp	120	0	13.5	0	0	Olive oil [2]	2	13	0.03
	Whole wheat bread - local bakery	1 slice	85	5	1	4	3	Bread [2]	1	30	0.03
	*Tomato	1 medium	22	4.78	0.25	1	1.48	Tomato [1]	0.48	70	0.03
	Butter - local dairy	1 tbsp	102	0	11.5	0.12	0	Butter [1]	7.3	14	0.10
**Snack**	Airly crackers - cheddar	1 serving	130	17	6	3	1	N/A	†see calc.	29	-0.00285
	Hummus - locally made	3 tbsp	105	6	7.5	3	1.5	Sauces, seasonings, condiments [2]	4	42	0.17
**Lunch**	*Carrots	1/2 cup	26	6	0	0.6	2	Carrots [2]	0.3	120	0.04
	Albacore canned tuna (local	1 pouch	120	0	4	21	0	Fish [2]	4	160	0.64

	pacific tuna)										
	Mayonnaise - locally made	1 tbsp	100	0	11	0	0	Sauces, seasonings, condiments [2]	4	30	0.12
	Whole wheat bread - local bakery	2 slices	170	10	2	8	6	Bread [2]	1	60	0.06
	*Romaine lettuce	1/4 bunch	5	1	0	0.4	0.5	Lettuce [1]	0.33	25	0.008
**Snack**	*Apple - locally grown - Fall season	1 medium	104	27.6	0.28	0.58	2.81	Apple [1]	0.36	154	0.06
	Peanut butter - ground in store	1 tbsp	94	3.5	8	3.7	1.3	Peanut butter, Peanut paste [1]	1.75	15	0.03
**Dinner**	Chicken breast - local free range, organic	0.5 breast	150	0	8.05	18.1	0	Chicken bone free meat [1]	3.07	75	0.23
	*Spinach	2 bunches	17	1.6	0	1.7	1	Spinaches [2]	0.2	60	0.01
	Olive Oil - in-state made	2 tbsp	240	0	27	0	0	Olive oil [2]	2	26	0.05
	*Zucchini	1 medium	33	6	0	2.4	2	Vegetables [2]	1	200	0.2
	Brown rice - locally grown	1/2 cup	124	26	1	2.25	1.6	Rice [1]	2.63	88	0.23
	Cucumber	1/4 cup	6	1.5	0	0	0.2	Cucumber [2]	3	40	0.12
	Carrots	1/4 cup	12.5	3	0	0.25	0.75	Carrot [2]	0.3	40	0.01
	Olive oil	1 tbsp	64	11	5	0	0	Olive oil [2]	2	13	0.03
	Broccoli	1 cup	25	6	0	2	3	Broccoli [1]	0.5	71	0.04

Table S6.1.2. Climatarian Meat (Higher Impact) Scenario Nutrition Overview

Nutrition [3]	Kcal	CHO	FAT	PRO	FIBER(CHO)
**Totals**	1991	128.48	123.08	72.19	34.79
Calories		513.92	1107.72	288.76	139.16
Percentages		25.81%	55.64%	14.50%	6.99%

Table S6.1.3. Climatarian Meat (Higher Impact) Scenario Carbon Footprint Totals

Carbon Footprint	Kg CO_2eq
Breakfast	0.65
Lunch	0.86
Snacks	0.30
Dinner	0.73
**Total**	**2.49**

Table S6.2.1. Climatarian Vegetarian (Lower Impact) Scenario Meal Plan Overview

Meal [3,10]	Item	Quantity	Kcal	CHO	FAT	PRO	FIBER	Food Item from Database: Petersson [1], Song [2]	CF (kg CO2eq/ kg food)	Food Weight (g)	Carbon Footprint (kg CO2eq)
**Breakfast**	**Eggs	2	233	12	22	2	10	Eggs [1]	4.46	100	0.45
	**Green onion	1/2 cup	11	2	0	0.34	0.6	Onion [2]	0.5	26	0.01
	Olive oil - in-state made	1 tbsp	120	0	13.5	0	0	Olive oil [2]	2	13	0.03

	Whole wheat bread - local bakery	1 slice	85	5	1	4	3	Bread [2]	1	30	0.03
	**Tomato	1 medium	22	4.78	0.25	1	1.48	Tomato [1]	0.475	70	0.03
	Butter - local dairy	1 tbsp	102	0	11.5	0.12	0	Butter [1]	7.3	14	0.10
**Snack**	Airly crackers - cheddar	1 serv	130	17	6	3	1	N/A	†see calc.	29	-0.00285
	Hummus - locally made	4 tbsp	140	8	10	4	2	Sauces, seasonings, condiments [2]	4	56	0.22
**Lunch**	**Carrots	1/2 cup	26	6	0	0.6	2	Carrots [2]	0.3	120	0.04
	Black bean patty	1 patty	110	13	4.5	9	4	Bean (pinto dried) [1]	0.73	130	0.09
	Mayonnaise - locally made	1 tbsp	100	0	11	0	0	Sauces, seasonings, condiments [2]	4	30	0.12
	Whole wheat bread - local bakery	2 slices	170	10	2	8	6	Bread [2]	1	60	0.06
	**Romaine lettuce	1/4 bunch	5	1	0	0.4	0.5	Lettuce [1]	0.325	25	0.01
**Snack**	**Apple - locally grown - Fall season	1 apple	104	27.6	0.28	0.58	2.81	Apple [1]	0.358	154	0.06
	Peanut butter - ground in	1 tbsp	94	3.5	8	3.7	1.3	Peanut butter,	1.75	15	0.03

	store							Peanut paste [1]			
**Dinner**	Tofu - firm	100g	85	1	4.19	10.9	0.9	Tofu [1]	1.072	100	0.11
	**Spinach	2 bunches	17	1.6	0	1.7	1	Spinaches [2]	0.2	60	0.01
	Olive oil - in-state made	2 tbsp	240	0	13.5	0	0	Olive oil [2]	2	26	0.05
	**Zucchini	1 zucchini	33	6	0	2.4	2	Vegetables [2]	1	200	0.2
	Brown rice - locally grown, organic	1/2 cup	124	26	1	2.25	1.6	Rice [1]	2.63	88	0.23
	Cucumber	1/4 cup	6	1.5	0	0	0.2	Cucumber [2]	3	40	0.12
	Carrots	1/4 cup	12.5	3	0	0.25	0.75	Carrot [2]	0.3	40	0.01
	Olive oil	1 tbsp	64	11	5	0	0	Olive oil [2]	2	13	0.03
	Broccoli	1 cup	25	6	0	2	3	Broccoli [1]	0.5	71	0.04

Table S6.2.2. Climatarian Vegetarian (Lower Impact) Scenario Nutrition Overview

**Nutrition [3]**	**Kcal**	**CHO**	**FAT**	**PRO**	**FIBER(CHO)**
**Totals**	1951	144.48	108.72	53.99	40.19
Calories		577.92	978.48	215.96	160.76
Percentages		29.03%	49.15%	10.85%	8.07%

Table S6.2.3. Climatarian Vegetarian (Lower Impact) Scenario Carbon Footprint Totals

**Carbon Footprint**	**Kg CO_2eq**
Breakfast	0.65
Lunch	0.32

Snacks	0.30
Dinner	0.60
**Total**	**1.87**

References:

1. Petersson, T.; Secondi, L.; Magnani, A. et al. A multilevel carbon and water footprint dataset of food commodities. *Sci Data* **2021**, *8*, 127. https://doi.org/10.1038/s41597-021-00909-8
2. Song, L.; Cai, H.; Zhu, T. Large-scale microanalysis of U.S. household food carbon footprints and reduction potentials. *Environmental Science & Technology* **2021**, *55*, 15323–15332. https://doi.org/10.1021/acs.est.1c02658
3. Food and Nutrition - U.S. Food and Drug Administration. Available online: https://www.usda.gov/topics/food-and-nutrition (accessed 11 October 2022).
4. MyPlate | U.S. Department of Agriculture. Available online: https://www.myplate.gov/ (accessed on 13 October 2022).
5. Estruch, R.; Ros, E.; Salas-Salvadó, J.; Covas, M.-I.; Corella, D.; Arós, F.; Gómez-Gracia, E.; Ruiz-Gutiérrez, V.; Fiol, M.; Lapetra, J.; Lamuela-Raventos, R. M.; Serra-Majem, L.; Pintó, X.; Basora, J.; Muñoz, M. A.; Sorlí, J. V.; Martínez, J. A.; Fitó, M.; Gea, A.; Martínez-González, M. A. Primary Prevention of Cardiovascular Disease with a Mediterranean Diet Supplemented with Extra-Virgin Olive Oil or Nuts. *NEJM* **2018**, *378*, e34. https://doi.org/10.1056/NEJMoa1800389
6. Hever J. Plant-based diets: A physician's guide. The Permanente journal 2016, 20, 15–082. https://doi.org/10.7812/TPP/15-082
7. Diet review: Paleo diet for weight loss. The Nutrition Source. Available online: https://www.hsph.harvard.edu/nutritionsource/healthy-weight/diet-reviews/paleo-diet/ (accessed on 18 October 2022).
8. Jönsson, T.; Granfeldt, Y.; Ahrén, B.; Branell, U.; Pålsson, G; Hansson, A.; Söderström, M.; Lindeberg, S. Beneficial effects of a Paleolithic diet on cardiovascular risk factors in type 2 diabetes: a randomized cross-over pilot study. Cardiovasc Diabetol 2009, 3, 1229–1232. https://doi.org/10.1186/1475-2840-8-35
9. Neudorf, H.; Mindrum, M.; Mindrum, C.; Durrer, C.; Little, J.P. A low-carbohydrate, high-fat ketogenic diet program implemented by an interdisciplinary primary care team improves markers of cardiometabolic health in adults with type 2 diabetes: A retrospective secondary analysis. Canadian Journal of Diabetes 2021, 46, 302–306. https://doi.org/10.1016/j.jcjd.2021.09.001
10. Climatarian – The easy, healthy, climate friendly diet. Available online: https://climatarian.com/ (accessed on 26 October 2022).
